# Bioinformatic Study of Transcriptome Changes in the Mice Lumbar Spinal Cord After the 30-Day Spaceflight and Subsequent 7-Day Readaptation on Earth: New Insights Into Molecular Mechanisms of the Hypogravity Motor Syndrome

**DOI:** 10.3389/fphar.2019.00747

**Published:** 2019-07-11

**Authors:** Maksim Sergeevich Kuznetsov, Artur Nicolaevich Lisukov, Albert Anatolevich Rizvanov, Oksana Victorovna Tyapkina, Oleg Aleksandrovich Gusev, Pavel Nicolaevich Rezvyakov, Inessa Benedictovna Kozlovskaya, Elena Sergeevna Tomilovskaya, Evgeny Evgenievich Nikolskiy, Rustem Robertovich Islamov

**Affiliations:** ^1^Department of Medical Biology and Genetics, Kazan State Medical University, Kazan, Russia; ^2^Institute of Fundamental Medicine and Biology, Kazan Federal University, Kazan, Russia; ^3^Kazan Institute of Biochemistry and Biophysics, Federal Research Center “Kazan Scientific Center” of RAS, Kazan, Russia; ^4^RIKEN Institute, Yokohama, Japan; ^5^Institute of Biomedical Problems of Russian Academy of Sciences, Moscow, Russia

**Keywords:** Bion-M1 biosatellite, 30-day spaceflight, 7-day postflight readaptation, mice lumbar spinal cord, hypogravity motor syndrome, transcriptome, bioinformatic study

## Abstract

The hypogravity motor syndrome (HMS) is one of the deleterious impacts of weightlessness on the human body in orbital space missions. There is a hypothesis that disorders of musculoskeletal system as part of HMS arise in consequence of changes in spinal motor neurons. The study was aimed at bioinformatic analysis of transcriptome changes in lumbar spinal cords of mice after a 30-day spaceflight aboard biosatellite Bion-M1 (space group, S) and subsequent 7-day readaptation to the Earth’s gravity (recovery group, R) when compared with control mice (C group) housed in simulated biosatellite conditions on the Earth. Gene ontology and human phenotype ontology databases were used to detect biological processes, molecular functions, cellular components, and human phenotypes associated with HMS. Our results suggest resemblance of molecular changes developing in space orbit and during the postflight recovery to terrestrial neuromuscular disorders. Remarkably, more prominent transcriptome changes were revealed in R vs. S and R vs. C comparisons that are possibly related to the 7-day recovery period in the Earth’s gravity condition. These data may assist with establishment of HMS pathogenesis and proposing effective preventive and therapeutic options.

## Introduction

Absence of gravity causes changes in virtually all organs and systems of a living organism at the molecular, cellular, and tissue levels (Edgerton and Roy, 2000). Fundamental knowledge of these changes extends our understanding of the human body functioning in the extreme microgravity environment of outer space and offers a clearer view of preventive options needed for astronauts on long missions.

Investigations of weightlessness effects on living organisms began with the launch of dog Laika to space in 1957 and the flight of Yuri Gagarin in 1961. Since then, studies in the domain of space medicine have revealed a variety of spaceflight effects on human cardiovascular, neurovestibular, and musculoskeletal systems (Grigoriev and Potapov, 2013). The hypogravity motor syndrome (HMS) is considered to be a severe microgravity effect on astronauts (Grigoriev and Kozlovskaya, 1991), which is why success of future remote space missions will be highly dependent on how soon we get to the roots of HMS pathogenesis and be ready to offer methods of prevention on the molecular level. An important input to this effort comes from experiments with animals that have been exposed to spaceflight weightlessness (Moyer et al., 2016).

Mechanisms of HMS development are still poorly explored. Interestingly, pathognomonic signs are observed in skeletal muscles following spinal or peripheral nerve disorders (Hides et al., 2017). Earlier, it has been shown that the important trigger of HMS is violation of sensory impulses from the skin (Kozlovskaya et al., 1981). Possibly, absence of sensory stimuli from mechanoreceptors of the sole skin results in the functional activity of motor neurons innervating leg muscles. However, it is well known that in a motor unit, the neuron and skeletal muscle fibers interrelate through electrical impulses and informative molecular signaling (Chevrel et al., 2006; Sakuma and Yamaguchi, 2011; Baguma-Nibasheka et al., 2016). Thus, skeletal muscle plasticity depends on the condition of the different inputs to motor units. In light of this assumption and based on our earlier studies of the neuromuscular synapse, peripheral nerve, and spinal cord in a rat model of hypogravity [hind limb unloading model (HUM)] (Chelyshev et al., 2014; Islamov et al., 2015), we made a supposition that HMS pathogenesis is invoked by spinal motor neurons.

Under the auspices of the Federal Space Program, the Russian Federal Space Agency and the Institute of Biomedical Problems of the Russian Academy of Sciences undertook a vast program of space bioresearches aboard biosatellite Bion-M1 launched on April 19 and landed on May 19, 2013 (Andreev-Andrievskiy et al., 2014). To test the hypothesis that changes in spinal motor neurons provoke HMS, a full genome study of the mice lumbar spinal cord was performed after the 30-day Bion-M1 mission. The list of genes from the lumbar spine of mice with significant increases and decreases has been presented in our previous study (Islamov et al., 2016). Some changes in gene expression supported our hypothesis that molecular changes in spinal motor neurons are among the key factors in HMS pathogenesis. Meanwhile, the further comparative bioinformatic analysis based on the contemporary genetic and medical databases is needed to discover the possible molecular mechanism of HMS development. At the same time, it is known that back on Earth, astronauts undergo lengthy recovery or rehabilitation (Loehr et al., 2015). To date, the problem of rapid rehabilitation of astronaut’s activity is under the intensive study. Obviously, the changes in the transcriptome profile arising during the readaptation to the Earth’s gravity may explain some clinical signs in astronauts after landing. The Bion-M1 research program included studies of mice immediately following the exposure to the 30-day spaceflight and after the 7-day readaptation on Earth. The chosen periods were designed for international collaborative investigation to obtain data on long-term exposure to weightlessness on varied physiological systems and provide data on behavioral readaptation of mice to Earth’s gravity after the flight (Andreev-Andrievskiy et al., 2014).

The investigation was designed as a full-genome study and bioinformatic analysis of transcriptome changes in the lumbar spinal cord of mice that spent 30 days onboard Bion-M1 in comparison with their counterparts that were given a week of rehabilitation to the Earth’s gravity. We examined transcriptome changes further using gene ontology (GO) and human phenotype ontology (HPO) databases for disclosure of the HMS pathogenesis and the relationship of HMS with the terrestrial neuromuscular diseases.

## Materials and Methods

### Animals and Treatment

Adult male C57BL/6J mice (4‒5 months of age, 25.1 ± 3.2 g) obtained from the “Puschino” animals breeding laboratory and nursery (Puschino, Moscow region, Russia) were divided in the flight and control groups. In its turn, the flight group was subdivided into the group of mice sacrificed 14 h following the biosatellite landing (space group, S) and the other one where the mice were given 7 days for readaptation (recovery group, R). Mice from the control (C) group were housed three per cage under simulated biosatellite conditions on Earth. Male mice were selected since they are physically stronger than female and have more stable hormonal status. The overview of the mice selection, housing, and training before they were launched, during the 30-day spaceflight and in postflight 7-day period of readaptation, was published by Andreev-Andrievskiy et al. (2014). The animal protocols including euthanasia were reviewed and approved by the Commission on Bioethics at the Institute of Mitoengineering of the Lomonosov Moscow State University (protocol no. 35 of November 1, 2012) and the Commission on Biomedical Ethics at the Institute of Biomedical Problems of the Russian Academy of Sciences (protocol no. 319 of April 4, 2013).

### Full-Genome Study of the Mice Lumbar Spinal Cord

In our investigation, biospecimens were obtained from six experimental animals. Lumbar spinal cords were harvested from groups S (n = 2), R (n = 2), and C (n = 2). The spinal cords of group C were extracted simultaneously with S group. Spinal cords have been removed from the vertebral column by hydraulic ejection method. Ventral and dorsal roots were sectioned, and L1 to L5 region of the lumbar enlargement were isolated and frozen in liquid nitrogen immediately. The prepared tissue samples were processed for RNA isolation from both the gray and white matter.

The microarray analysis procedures for group R were performed as described previously for groups S and C (Islamov et al., 2016). In short, total RNA was extracted from the entire sample of lumbar enlargements using the RNeasy Mini Kit (Qiagen, Valencia, CA). RNA quality was confirmed with 2100 Bioanalyzer (Agilent Technologies, Santa Clara, CA) where 18S and 28S ribosomal RNA served as controls. For microarray analysis, 500 ng of RNA in the Mouse GE 4x44K v2 Microarray Kit (Agilent Technologies, Santa Clara, CA) was used. Microarray analysis procedures were conducted as per manufacturer’s instructions. Quality control of the feature was performed using the settings recommended by Agilent Technologies. Raw microarray data were uploaded in ArrayExpress (accession: E-MTAB-7426).

### Bioinformatic Study

We used R version 3.4.4 (R Foundation for Statistical Computing, Vienna, Austria) for all steps of data processing and analysis (code is available on github.com/bqmax/bion_m1) (R Core Team, 2018). Background correction and quantile-based normalization were performed before removal of control probes and within-array replicates replacement with averages. We used the hierarchical clustering with complete agglomeration and Euclidean distance as a similarity measure and principal component analysis to assess general transcriptome profiles of the experimental spinal cords (Aziz et al., 2017). Linear models implemented in limma package were applied to assess differential genes expression. The Benjamini–Hochberg procedure was used to control the false discovery rate (Ritchie et al., 2015).

To determine differentially expressed genes, the followed cutoff rules were applied: p_FDR_-value <0.05 and absolute log2(fold change) > 2 (or absolute fold change >4).

For functional analysis of the obtained microarray data, we used two databases, namely GO and HPO as follows:

1. Genes sets considered as differentially expressed were selected for GO-based and HPO-based overrepresentation analysis *via g:Profiler interface* (Reimand et al., 2016);
2. Random forest algorithm implemented in GOexpress package was used for GO-based enrichment analysis (Rue-Albrecht, 2016).

### Real-Time PCR Analysis

Transcriptome data were validated by real-time PCR (RT-PCR) using SYBR Green method. Differentially regulated genes (Echs1, Ndufa9, Rplp2, Fbxo21, and Parl) were selected by both minimization of observed p-values and effect sizes (absolute fold changes). Ppib and Rpl7 genes were chosen as housekeeping references due to stable expression in different mouse tissues (Thomas et al., 2014; Bruckert et al., 2016). Primers with appropriate thermodynamic properties for these genes were designed using Vector NTI software (Invitrogen); specificity of these primers was confirmed using BLASTn. Random Hexamers (Trermo Fisher) and RevertAid transcriptase (Thermo Scientific) for reverse transcription and qPCRmix-HS SYBR (Eurogene) for quantitative PCR were employed. For RT-PCR data analysis, REST software (QIAGEN, GmbH) was used. The list of the target genes and corresponding sequences of designed primers are presented in **Table S1**.

## Results

### Hierarchical Clustering and Principal Component Analysis

**Figure 1:**
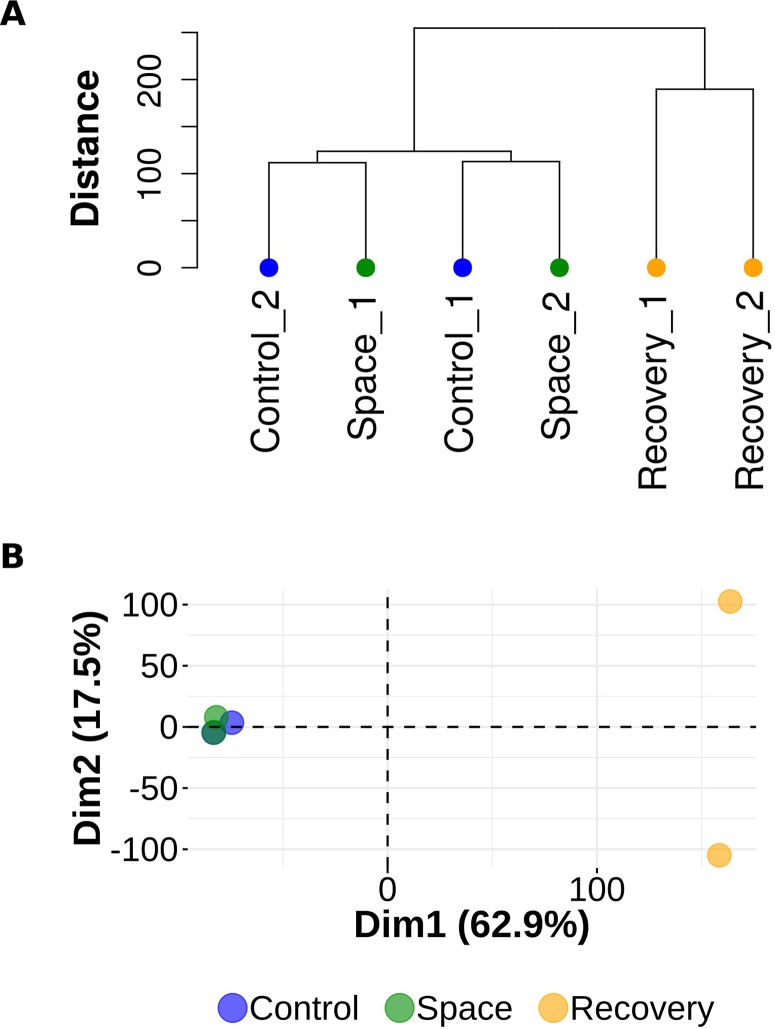
Hierarchical clustering and principal component analysis. **(A)** Dendrogram representing hierarchical clustering of gene expression data. Observations from the R (recovery) group form a single cluster. Observations from the S (space group) and C (control) groups are in a compound cluster. **(B)** Principal component analysis of gene expression data. Observations from the S (space) and C (control) groups are mixed in PC1 (Dim 1) and PC2 (Dim 2) spaces.

Experimental observations form two distinct clusters, one of which consists of two samples from group R and the other is a compound of observations from groups S and C. Dendrogram on **Figure 1A** illustrates the arrangement of clades produced by hierarchical clustering. Similar results obtained with principal component analysis are visualized on the biplot (**Figure 1B**).

### Microarray Expression Profiling

In our previous research, we employed the Mouse GE 4x44K v2 Microarray Kit to perform transcriptome analysis of the lumbar spinal cord of mice flown on Bion-M1. In comparison with group C, out of genes declared on the microarray platform increased and decreased expressions were documented in 134 genes and 41 genes, respectively. The list of up- and downregulated genes is published by Islamov R. and coauthors (Islamov et al., 2016).

In the present study, the same Mouse GE 4x44K v2 Microarray Kit was used for transcriptome profiling of the lumbar spinal cord from mice after 7 days of readaptation after the Bion-M1 mission (group R).

**Figure 2:**
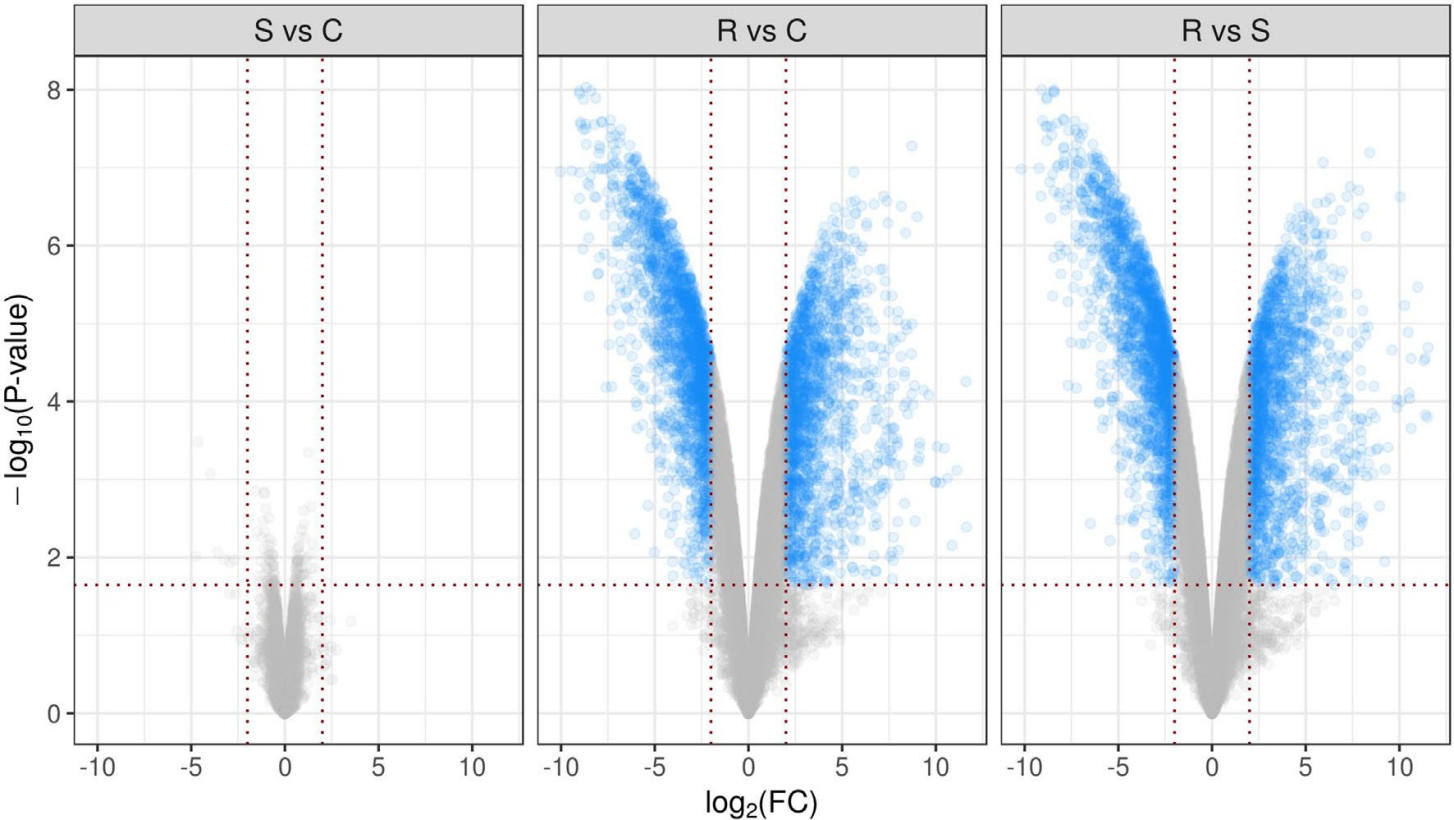
Volcano plots present differential performance of gene expression in mice lumbar spinal cord in three comparisons. Sets of genes considered as differentially expressed using declared cutoff rules [p_FDR_-value <0.05 and absolute log_2_(Fold change) >2] are marked as blue dots. -log_10_ of observed p-values are mapped to the y axis.

**Figure 3:**
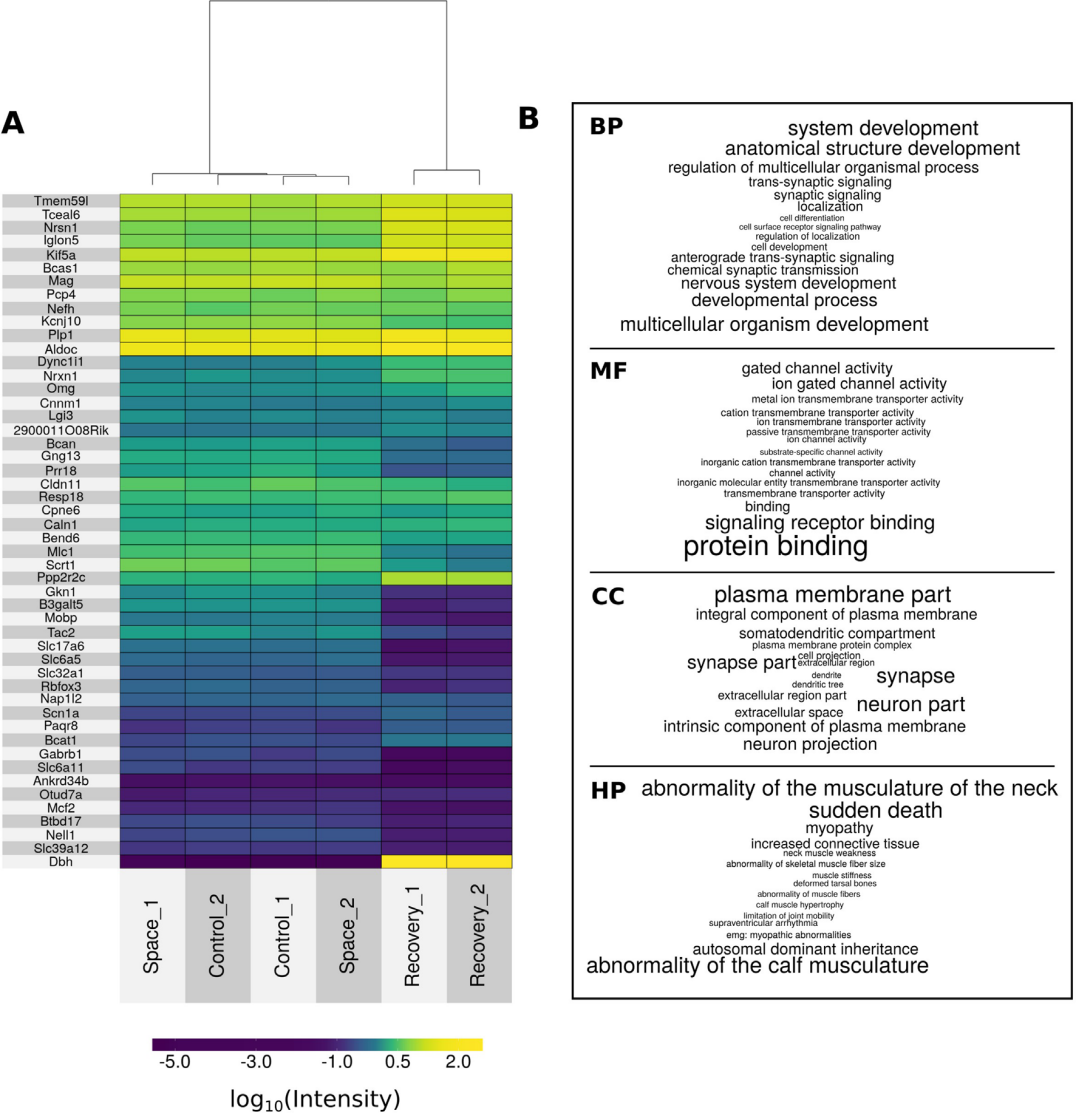
The top of differentially expressed genes and GO terms. **(A)** Heat map of top 50 differentially expressed genes after hierarchical biclustering. **(B)** Word clouds of top 15 enriched GO terms from biological processes (BP), molecular functions (MF), and cellular components (CC) namespaces and top 15 enriched HPO terms (HP). Letter size of words is proportional to 1/-log_10_(p_FDR_-value).

Comparative analysis between groups R and C using the declared cutoff rules revealed 1,994 downregulated and 1,726 upregulated genes. In comparison between R and S groups, there were 2,025 downregulated and 1,758 upregulated genes. For clarity, differential performance of gene expression in mice lumbar spinal cord in three comparisons is presented in the form of volcano plots (**Figure 2**). It is important to note that multiple groups comparison results in decrease of statistical power which did not reveal differentially expressed genes between S and C groups, as was shown earlier (Islamov et al., 2016). Full lists of genes considered as differentially expressed with appropriate statistics are presented in **Table S2**. **Figure 3A** represents top 50 differentially expressed genes after hierarchical biclustering.

### Functional Profiling of Gene List Using g:Profiler

Based on the full list of differentially expressed genes, GO-based and HPO-based overrepresentation analysis was performed. **Table S3** contains all overrepresented GO:biological processes (n = 1,775), GO:molecular functions (n = 321), GO:cellular components (n = 236), and HPO terms (n = 43). **Figure 3B** represents top 15 overrepresented HPO and GO terms based on p_FDR_-value. Notably, top-rated biological processes terms are semantically associated with the nervous system and are consistent with revealed molecular functions, cellular components, and human phenotypes.

### Gene Ontology-Based Enrichment Analysis

**Figure 4:**
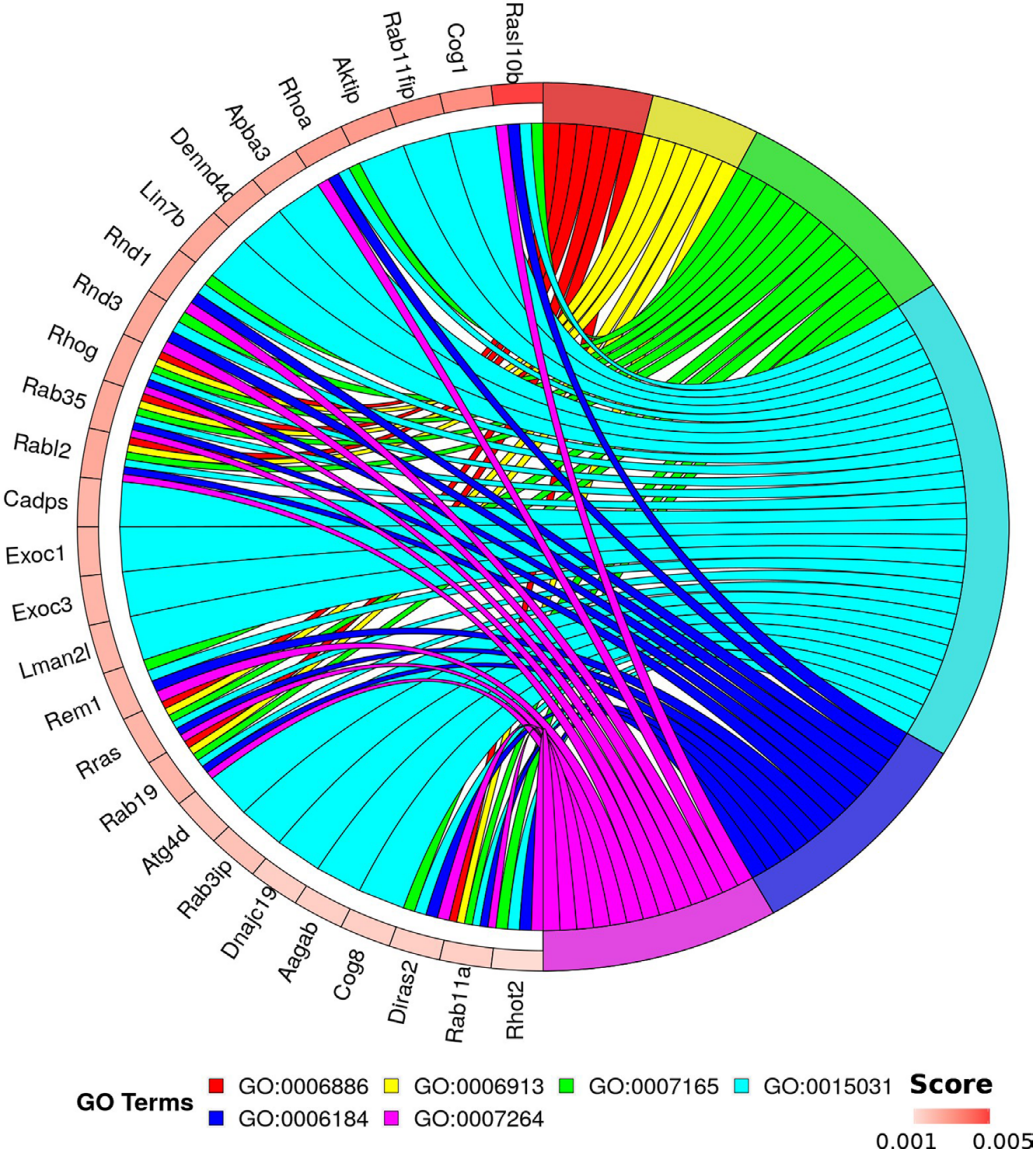
Genes and GO terms forming “hot spot” of enrichment. Top rated 1,000 genes (based on Gini index) and enriched GO terms from biological processes (p < 0.01) were selected for “hot spot” detection. Circular visualization represents links between 28 genes and six terms. Genes are scored by Gini index. GO:0006886—intracellular protein transport; GO:0006913—nucleocytoplasmic transport; GO:0007165—signal transduction; GO:0015031—protein transport; GO:0006184—obsolete GTP catabolic process; and GO:0007264—small GTPase mediated signal transduction.

This analysis allows discrimination of biological processes, molecular functions, and cellular components that are possibly involved with pathological processes in the spinal cord in the condition of weightlessness without loss of information about expression levels of genes in each experimental observation. The results of functional enrichment based on random forest are shown in **Table S4**. Ranking of enriched GO terms depends on random forest results (aggregation of Gini scores for each genes linked with term) and count of mapped genes. For downstream analysis, we selected the top 1,000 genes based on Gini score and only terms from biological processes namespace with p-value <0.01 and total genes count ≥10. Using binary biclustering, we identified “hot spots” of enrichment by bivariate maximization (implemented algorithm and R code are described in **Appendix S1**). The results of analysis are presented in **Figure 4**. This approach allowed to reveal 28 genes linked with six GO terms: GO:0006886 (intracellular protein transport); GO:0006913 (nucleocytoplasmic transport); GO:0007165 (signal transduction); GO:0015031 (protein transport), GO:0006184 (obsolete GTP catabolic process), and GO:0007264 (small GTPase mediated signal transduction).

### Real-Time PCR Validation of Microarray Data

The RT-PCR results have confirmed the directions of the obtained transcriptome changes in five of five cases in R vs. S comparison and in four of five cases in S vs. C comparison (gene Parl was upregulated in RT-PCR assay and downregulated in microarray approach). **Table S1** contains fold changes for five target genes estimated in both microarray and RT-PCR analysis. It should be noted that our results are consistent with the study on the reliability of the use of RT-PCR for verifying the microarray data (Morey et al., 2006).

## Discussion

Spaceflight and exposure to microgravity cause specific changes in human skeleton (Smith et al., 2012) and skeletal muscles (Belavý et al., 2011; Narici and de Boer, 2011). It is known that the disorders seen in astronauts after a space mission are similar to terrestrial neuromuscular diseases in patients (Hides et al., 2017). Studies of the neuromuscular system plasticity in astronauts are beneficial to patients with similar disorders, and, vice versa, researches with patients may provide new options for the reconditioning of astronauts (Stokes et al., 2017). HMS is among the most untoward consequences of long-term orbital spaceflight. HMS is characterized by specific changes in skeletal muscles, particularly in the so-called postural muscles responsible for maintaining posture in the gravity field of the Earth (Grigoriev and Kozlovskaya, 1991). Along with weakness and atrophy of skeletal muscles, the ratio of muscle fiber types, expression of muscle-specific proteins, contractile characteristics, and electrophysiological properties of skeletal muscle fibers are affected gravely (Shenkman, 2016). The physiotherapy methods applied to patients on Earth and experience of spaceflights has shown that the most effective way to maintain an astronaut’s capacity for work and to be prepared to return to Earth is regular performance of a complex set of physical exercises (Grigoriev and Kozlovskaya, 1991) that astronauts have to accomplish several hours a day in order to prevent HMS (Loehr et al., 2015).

The proposed role of spinal motoneurons in HMS pathogenesis requires a deep insight into the molecular and cellular disorders in the spinal cord. However, up to date, little is known about microgravity effects on the central nervous system. Few reports on spinal cord of rodents after different periods of orbital flight are available. Thus, it was shown that succinate dehydrogenase activity was selectively decreased in the medium-sized motoneurons of the mice lumbar spinal cord after the 9-day spaceflight (Ishihara et al., 2006). In rat spinal motoneurons, the content of cytoplasmic proteins was significantly lowered 22 days after spaceflight (Gorbunova and Portugalov, 1976). Signs of myelin destruction and decreased number of myelin-forming cells in white matter (Povysheva et al., 2016) and changes in immonoexpression of choline acetyltransferase (ChAT) and neurofilament proteins in gray matter (Porseva et al., 2016) were shown in mice spinal cord after the 30-day exposure to microgravity. During the longest mission in space, which lasted 91 days, unfortunately, only three mice survived, and only data on spinal cord necropsy was presented (Cancedda et al., 2012). In general, these results demonstrate the evidence of negative effects of weightlessness on spinal cord, which may lead to HMS, although the particular mechanisms of HMS pathogenesis still need to be fully elucidated. In our investigation, we studied lumbar spinal cord housing motoneurons that innervate hind limb skeletal muscles having an antigravity function.

The contemporary “omics” technologies are widely employed in unveiling the mechanisms of human diseases. Microarray data analysis makes it possible to examine the transcriptome changes at the cell, tissue, or organic levels (Ewis et al., 2005). Numerous bioinformatics studies on gene expression profiling in the spinal cord of mice with neurotrauma or neurodegenerative diseases have been performed in recent years (Munro et al., 2012; Elliott et al., 2013; Zhu et al., 2017; Barham et al., 2018). There have been only few studies of spaceflight effects on the transcriptome profile in the bone tissue, immune system, and skeletal muscles of mammals (Nichols et al., 2006). A substantial progress in microgravity genomics has been made owing mostly to the investigations of rodent skeletal muscles. For instance, Gambara and coauthors obtained a global gene expression profile of the paraspinal skeletal muscle (longissimus dorsi) (Gambara et al., 2017a) as well as the slow-twitch (soleus) and fast-twitch (extensor digitorum longus) hind limb muscles (Gambara et al., 2017b) following the exposure onboard spacecraft Bion-M1. It was shown that microgravity strongly affected the transcriptome profile in the postural soleus muscle and slightly changed the gene expression pattern in the extensor digitorum longus and longissimus dorsi. These data pointed to the microgravity-sensitive muscle genes involved in pathogenesis of HMS.

In this study, for the first time, we investigated lumbar spinal transcriptomes of mice after their 30-day spaceflight on biosatellite Bion-M1 and subsequent 7-day readaptation on Earth. Transcriptome analysis of the obtained data was completed in three comparisons, i.e., spaceflight (S) vs. ground control (C), 7-day postflight recovery (R) vs. C, and R vs. S. The results that were received with the involvement of the GO and HPO databases suggest that molecular changes developed in the mice lumbar spinal cord during the flight are similar to those in consequence of terrestrial neuromuscular disorders (Hides et al., 2017; Stokes et al., 2017). Thus, discovered biological processes terms (nervous system development, synaptic signaling, and anterograde trans-synaptic signaling), molecular functions (signaling receptor binding, gated channel activity, and ion gated channel activity), and cellular components (plasma membrane part, synapse, and neuron projection) are highly linked with human phenotypes (electromyography: myopathic abnormalities, abnormality of muscle fibers, muscle stiffness), which are in line with signs of HMS.

Earlier, using the mice HUM, we hypothesized that HMS pathogenesis partly may be due to spinal motoneurons disorders (Islamov et al., 2011; Chelyshev et al., 2014). Taking into consideration our HUM findings demonstrating decreases in the gray and white matter areas, decrease in ChAT immunoexpression, changes in myelin gene expression, and phenotypic modifications of glial cells in lumbar spinal cords, the results of this investigation suggest that motoneurons contribute to the HMS development. These findings are in a very good agreement with the data obtained in the present bioinformatic analysis. The discovered GO-based biological processes, molecular functions, and cellular components in spinal cord support conclusions in our previous report (Povysheva et al., 2016) that demyelination in the central nervous system is a factor in the HMS development. Moreover, it is notable that discovered GO:0006886 (intracellular protein transport), GO:0006913 (nucleocytoplasmic transport), and GO:0015031 (protein transport) may be associated with the axonal transport. It is known that axonal proteins are synthesized in the motor neuron perikaryon and then are distributed over the axon by the mechanism of anterograde axonal transport. The distance to which the molecules are transported varies significantly. In fact, the length of human neural outgrowths may by more than 1 m. Thus, to our knowledge, this is the first report that molecular disorders in intracellular transport system may affect the axonal transport that may be one of the important mechanism of HMS pathogenesis. Furthermore, overrepresented GO terms based on differentially expressed genes have revealed biological processes and molecular functions that are involved in synaptic plasticity (chemical synaptic transmission and synaptic signaling), cell membrane permeability (ion channels, potassium channel activity, and voltage-gated ion channel activity), and cytoskeleton (cytoskeletal protein binding and actin binding) and may be the key factors in the HMS pathogenesis as well (**Table S3**). These findings suggest resemblance of molecular changes developing in space and during the postflight recovery to the HPO terms for terrestrial neuromuscular disorders.

Under the auspices of the Bion-M1 program, we also used the immunohistochemical assay to investigate reactions of lumbar motor neurons from the S, R, and C groups (Tyapkina et al., 2016). It should be noted that the decreased immunoexpression of synaptic proteins (synaptophysin and postsynaptic density protein 95) in motor neurons of mice after the spaceflight (groups S and R) is consistent with more than 15-fold upregulation of the corresponding genes (Syp and Dlg4) in group R. Moreover, Porseva et al. (2016) showed that the number of neurons containing ChAT and neurofilament proteins decreased in the thoracic section of the spinal cord in mice after the 30-day spaceflight (group S in our research). According to our results, in a week after landing (group R), the level of ChAT gene revealed a 17-fold increase; levels of Nefl, Nefm, and Nefh genes increased 176, 284, and 176 times, respectively. Changes in the level of proteins in motoneurons resulted, possibly, in increased expression of the gravity-sensitive genes during the readaptation period. Meanwhile upregulation or downregulation of certain genes in spinal cord tissue after 7-day readaptation period may be due to not only compensatory reaction to 30-day period of disuse of musculoskeletal system in space but also activity of the microgravity-sensitive genes and their hierarchical status in specific biological processes. Our results are also in line with the findings of Gambara et al. (2017a) demonstrating expression of microgravity-sensitive non-muscle-specific genes that match with genes and trends in expression identified in the corresponding groups in our study.

Thus, the bioinformatic analysis of transcriptome changes presented in the study provides a molecular evidence of HMS resemblance to the pathogenesis of the terrestrial neurological disorders. However, because of a significant loss of mice during the mission, we received very few animals (n = 2 in each group) for our study (Andreev-Andrievskiy et al., 2014) and, therefore, were unable to provide an acceptable power to identify the entire pool of neuron-specific target genes. At the same time, a vast majority of transcriptomic studies (microarray and RNA-seq) are conducted with a small sample size that makes them underpowered and exploratory. In our study, linear models realized in limma package to determine differentially expressed genes were used. The implemented analysis can be considered as de facto standard approach, especially in case of small sample sizes and multiple group comparison (Gentleman et al., 2005). This being so, we reason that our results should be considered as exploratory due to the insufficient strength of evidence.

For today, the longest continuous presence of human in space amounts to 438 days. This period is comparable with the estimated time of a Martian mission that includes the transits to Mars and back to Earth and a short stay on the planet. As the tasks facing astronautics become more challenging, this period is likely to extend. Still, even diligent pursuance of expressly developed preventive complexes may fail to preclude HMS development completely. According to the Bion-M1 program, the mice were launched for a 30-day stay in orbit. However, major disorders in the musculoskeletal system develop in the first 2 weeks of spaceflight (Akima et al., 2000; LeBlanc et al., 1995). Therefore, the 30-day period of exposure to microgravity may be appropriate for discovery of tissue-specific and gravity-sensitive genes and intracellular pathways involved in the HMS development. Severe functional impairment of postural and locomotor musculature was obtained in mice after the 30-day spaceflight on the board Bion-M1 biosatellite (Andreev-Andrievskiy et al., 2014). These independent findings are in line with GO biological processes [locomotion (GO:0040011), locomotory behavior (GO:0007626), regulation of locomotion (GO:0040012), and musculoskeletal movement (GO:0050881)] terms and HPO terms [myopathy (HP:0003198) and proximal muscle weakness (HP:0003701)] revealed in our work. We believe that our bioinformatics study will help future experiments aimed at disclosure of the HMS pathogenesis and suggest advanced methods of preventing and treatment of HMS and the similar terrestrial neuromuscular disorders.

## Conclusion

Comprehensive bioinformatic analysis of genes expression profiling in the mice lumbar spinal cord after the 30-day spaceflight with subsequent 7-day recovery revealed molecular cascades that may be involved in pathogenesis of HMS. These data may assist in unveiling HMS pathogenesis and development of novel effective preventive and therapeutic options. Moreover, it was shown that postflight readaptation is complicated with further molecular changes in the condition of normal gravity. However, it is necessary to take into consideration that the identified genes and pathways probably associated with HMS development may be triggered not only by weightlessness but also accelerations during spacecraft insertion and descent, exposure to space radiation, or attenuation of the Earth’s magnetic field.

## Ethics Statement

The animal protocols including euthanasia were reviewed and approved by the Commission on Bioethics at the Institute of Mitoengineering of the Lomonosov Moscow State University (Protocol No. 35 of November 1, 2012) and the Commission on Biomedical Ethics at the Institute of Biomedical Problems of the Russian Academy of Sciences (Protocol No. 319 of April 4, 2013).

## Author Contributions

EN and RI contributed to the study conception and design. AL, PR, OT, and OG contributed to the acquisition of data. MK, AL, AR, and RI contributed to the analysis and interpretation of data. MK, AL, PR, and RI contributed to the drafting of the manuscript. AR, IK, ET, and RI provided critical revision.

## Funding

The study was funded by grant RFBR 17-04-00385, grant of Presidium of the Russian Academy of Sciences “Fundamental research for biomedicine technology development.” Albert Rizvanov was personally supported as a “leading scientist” by state assignment 20.5175.2017/6.7 of the Ministry of Science and Higher Education of Russian Federation. 

## Conflict of Interest Statement

The authors declare that the research was conducted in the absence of any commercial or financial relationships that could be construed as a potential conflict of interest.

The reviewer, LN, declared a shared affiliation, though no other collaboration, with three of the authors, OT, EN, and RI, to the handling Editor at the time of review.

## Supplementary Material

Table S1 Validation of selected differentially regulated genes in mice lumbar spinal cord in S (space) vs. C (control) and R (recovery) vs. S (space) comparisons.

Table S2 Differentially expressed genes identified by comparison between the R (recovery) and C (control) groups and between the R (recovery) and S (space) groups.

Table S3 Overrepresented GO and HPO terms based on differentially expressed genes with appropriate statistics.

Table S4 GO terms enriched using random forest with Gini feature scoring.

Data Sheet S1 R code for binary biclustering and genes/GO terms selection.
