# Supplementary material for: Bioinformatic Study of Transcriptome Changes in the Mice Lumbar Spinal Cord After the 30-Day Spaceflight and Subsequent 7-Day Readaptation on Earth: New Insights Into Molecular Mechanisms of the Hypogravity Motor Syndrome
Source: Front Pharmacol. 2019 Jul 11;10:747. doi: 10.3389/fphar.2019.00747 (PMC6637859; doi:10.3389/fphar.2019.00747)
Supplement: Data Sheet S1 — R code for binary biclustering and genes/GO terms selection. [file DataSheet_1.pdf]

GO terms and genes selection

Input:

Results of random forest algorithm and permutations implemented in GOexpress package – `pVal` object

Adjacency matrix and it's heatmap representation

Adjacency matrix was builded using mapping attribute from output object after random forest algorithm implementation

```
bp <- subset_scores(result = pVal,
                    namespace = "biological_process",
                    total = 10,
                    p.val=0.01)
m <- table(bp$mapping$gene_id, bp$mapping$go_id)
attributes(m)$class <- 'matrix'
```

Filtering 1000 most ranked genes linked mapped to 'Biological processes' namespace

```
m <- m[rownames(bp$genes)[1:1000], ]
```

Heatmap representation of matrix

```
m <- m[rownames(bp$genes)[1:1000], ]
superheat(m, legend = F)
```

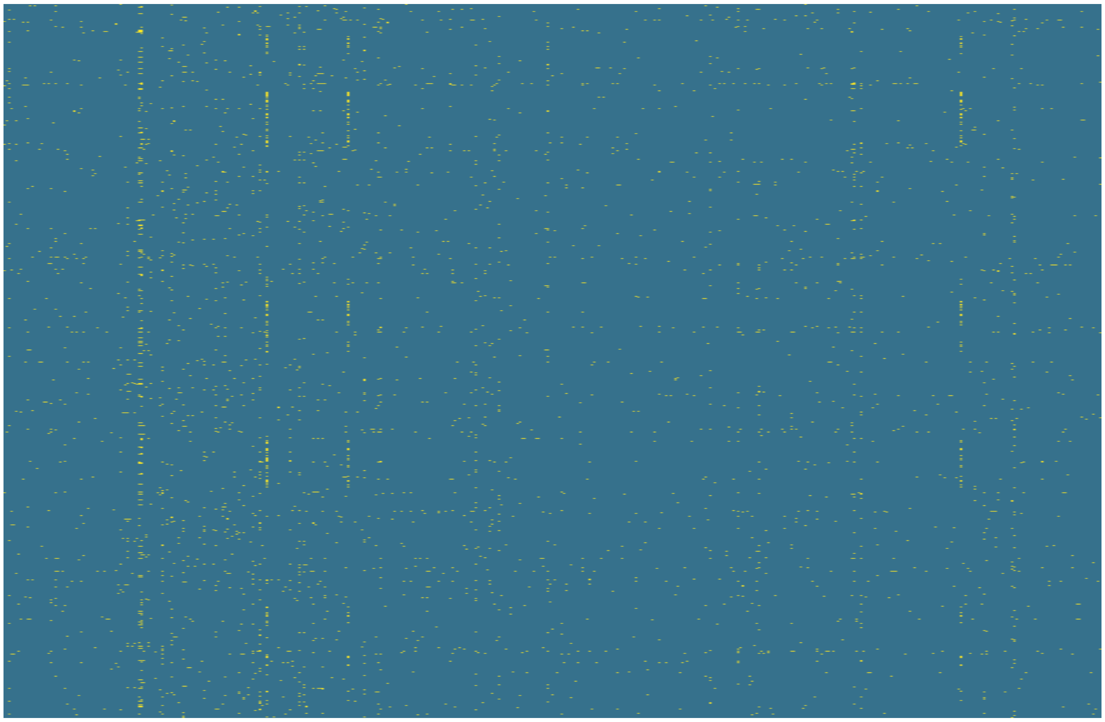

Binary clustering of rows and columns

```
a <- hclust(dist(t(m), method = 'binary'))
b <- hclust(dist(m, method = 'binary'))
```

Reordering of rows and columns of matrix

```
m <- m[b$order, a$order]
```

Heatmap representation of matrix after binary clustering and reordering

```
superheat(m, legend = F)
```

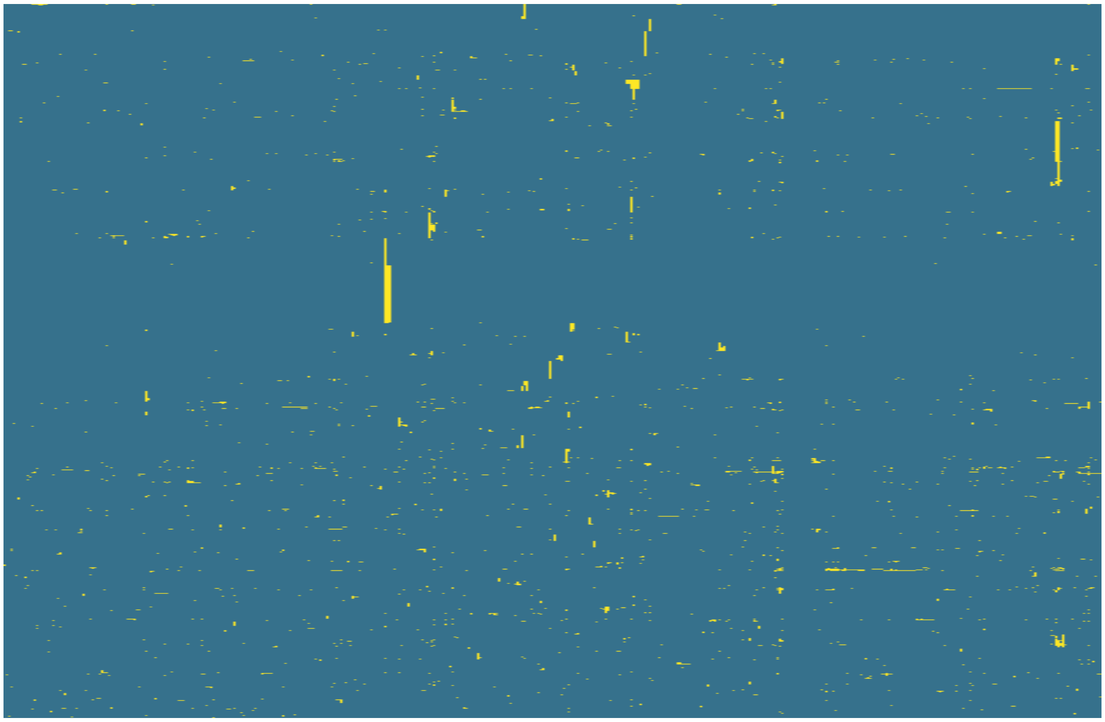

In the heatmap “hot spots” of enrichment shown in yellow. In the study we have chosen the „hot spot“ with maximal count of genes and maximal count of corresponding GO terms (bivariate maximum).

```
t <- m[867:894, 269:274]

superheat(t, legend = F,
          bottom.label.text.size = 3,
          bottom.label.text.angle = 90,
          left.label.text.size = 3,
          left.label.text.angle = 0,
          bottom.label.size = 0.2,
          left.label.size = 0.6,
          padding = 0.2)
```

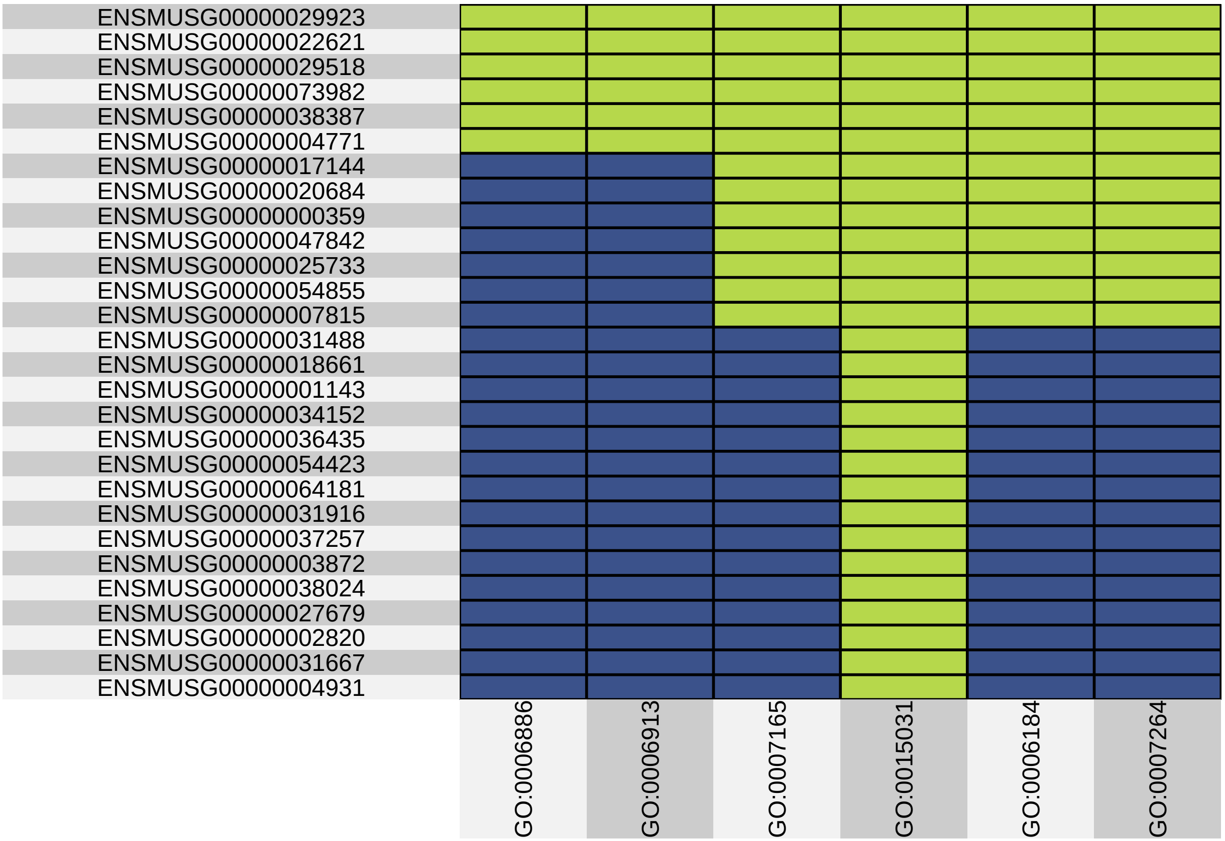

Circular visualization of results

```
rownames(t) <- mapIds(org.Mm.eg.db, keys = rownames(t), column = "SYMBOL",
                      keytype = "ENSEMBL", multiVals = 'first')
GOChord(t, gene.size = 4, nlfc = 0)+
    theme(legend.text = element_text(size = 13),
          legend.title = element_text(size = 15, face = 'bold'))
```

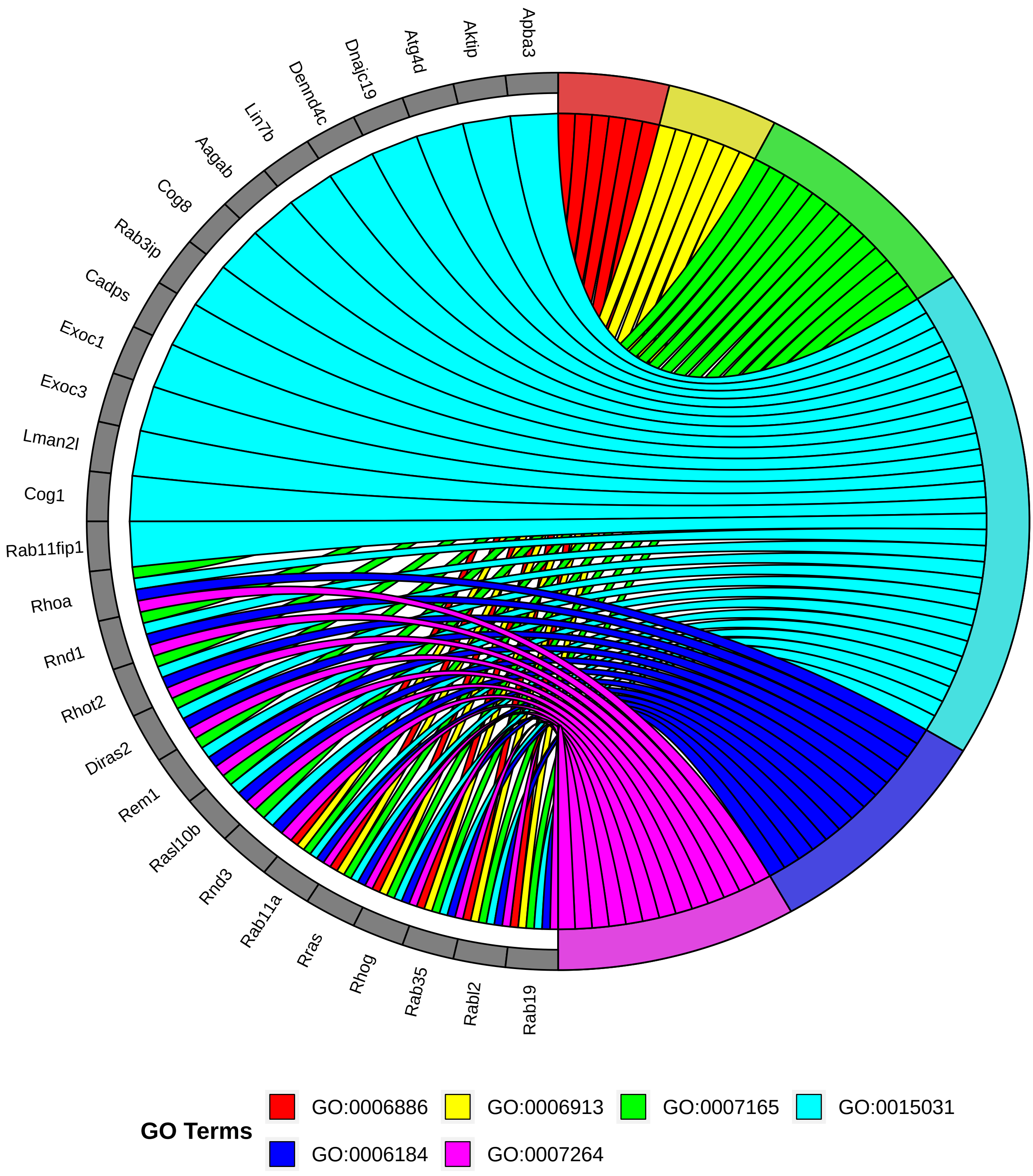
Apba3
Aktip
Atg4d
Dnajc19
Dennd4c
Lin7b
Aagab
Cog8
Rab3ip
Cadps
Exoc1
Exoc3
Lman2l
Cog1
Rab11fip1
Rhoa
Rnd1
Rhot2
Diras2
Rem1
Rasl10b
Rnd3
Rab11a
Rras
Rhog
Rab35
Rabl2
Rab19
GO Terms
GO:0006886
GO:0006913
GO:0007165
GO:0015031
GO:0006184
GO:0007264
